# Evaluating Efficiencies of Dual AAV Approaches for Retinal Targeting

**DOI:** 10.3389/fnins.2017.00503

**Published:** 2017-09-08

**Authors:** Livia S. Carvalho, Heikki T. Turunen, Sarah J. Wassmer, María V. Luna-Velez, Ru Xiao, Jean Bennett, Luk H. Vandenberghe

**Affiliations:** ^1^Grousbeck Center for Gene Therapy Boston, MA, United States; ^2^Schepens Eye Research Institute/Massachusetts Eye and Ear Infirmary Boston, MA, United States; ^3^Harvard Medical School Boston, MA, United States; ^4^Department of Urology, Radboud Institute for Molecular Life Sciences Nijmegen, Netherlands; ^5^Department of Ophthalmology, University of Pennsylvania School of Medicine Philadelphia, PA, United States

**Keywords:** AAV, dual AAV, retina, gene therapy, vector reconstitution, oversized AAV

## Abstract

Retinal gene therapy has come a long way in the last few decades and the development and improvement of new gene delivery technologies has been exponential. The recent promising results from the first clinical trials for inherited retinal degeneration due to mutations in *RPE65* have provided a major breakthrough in the field and have helped cement the use of recombinant adeno-associated viruses (AAV) as the major tool for retinal gene supplementation. One of the key problems of AAV however, is its limited capacity for packaging genomic information to a maximum of around 4.8 kb. Previous studies have demonstrated that homologous recombination and/or inverted terminal repeat (ITR) mediated concatemerization of two overlapping AAV vectors can partially overcome the size limitation and help deliver larger transgenes. The aim of this study was to investigate and compare the use of different AAV dual-vector approaches in the mouse retina using a systematic approach comparing efficiencies *in vitro* and *in vivo* using a unique oversized reporter construct. We show that the hybrid approach relying on vector genome concatemerization by highly recombinogenic sequences and ITRs sequence overlap offers the best levels of reconstitution both *in vitro* and *in vivo* compared to *trans*-splicing and overlap strategies. Our data also demonstrate that dose and vector serotype do not affect reconstitution efficiency but a discrepancy between mRNA and protein expression data suggests a bottleneck affecting translation.

## Introduction

Recently, the first ever adeno-associated virus (AAV) gene transfer therapeutic, Glybera®, was approved for commercialization in Europe for the treatment of familial lipoprotein lipase deficiency (LPLD) (www.uniqure.com/). Long-term evaluation of this therapy showed a reduction in the severity and incidence of pancreatitis (Gaudet et al., 2016). In the eye, advances in gene therapy are marked by the successful outcomes of clinical trials for Leber Congenital Amaurosis (LCA) (Bennett et al., 2016) and choroideremia (MacLaren et al., 2014). In these studies, patients had improved and sustained retinal sensitivity and LCA patients also had improved navigation in mobility tests under various light conditions, as a measure of how these treatments can improve the quality of life for patients (MacLaren et al., 2014; Bennett et al., 2016). These are only three of the 91 currently listed AAV mediated gene therapy clinical trials (https://clinicaltrials.gov/). The large number of on-going trials highlights the impact that gene transfer has on changing the way autosomal recessive diseases are treated.

Some of the main reasons to employ AAV for clinical therapies are that it has very low incidence of host genome integration, is well tolerated in most tissues, and production generates titer high enough to be used for humans (Ferreira et al., 2014). However, one of the main limiting factors of this strategy is the restriction of transgene size that can be packaged into AAV, which is ~4.8 kb (Wu et al., 2010). A study by Wu et al. (2010) showed that packaged AAV genomes never exceeded 5.2 kb in length, but overpackaging results in genome fragmentation. It has also been shown that vectors carrying larger genomes have lower transduction capacity, most likely due to a preferential degradation of vectors encapsidating larger genomes (Grieger and Samulski, 2005). Even though protein expression from oversized AAV vectors with fragmented genomes due to reconstitution of overlapping fragments has been detected (Grieger and Samulski, 2005), use of heterogenous fragmented vector preparations is not a feasible option in clinical applications. As a result, developing vectors to treat many retinal diseases, such as Usher's Syndrome Type 2A, which is caused by a mutation in a gene with a 15.8 kb coding region, is virtually impossible. Moreover, regulatory sequences that may be required to drive gene expression add to the final size and may render many genes too large for AAV packaging. Consequently, there is great interest in increasing transgene size while still utilizing the AAV system and the use of a dual AAV vectors system has been extensively studied for different experimental and disease modalities (Chamberlain et al., 2016).

The dual method to increase the size of the gene delivered to target cells consists in administering two separate gene fragments within independent AAVs. For this approach to work, the packaged transgenes must contain one of the following: (1) overlapping sequences to allow for homologous recombination, known as overlapping technique, (2) splice acceptor (sA) and splice donor (sD) sequences for intermolecular concatamerization and splicing in the *trans*-splicing technique, or (3) a hybrid of both of the above using a highly recombinogenic sequence known as the hybrid technique (Duan et al., 2001). It has been shown that the fragments will reassemble when transferred to a cell and produce a full-length protein (Yan et al., 2000, 2002; Duan et al., 2003; Hirsch et al., 2009). In the retina, the dual approach was first reported by Reich et al. (2003) which used the *trans*-splice technique to show that β-galactosidase expression *in vivo* was around 40% after AAV5 subretinal injection (Reich et al., 2003). Furthermore, the three dual approaches (*trans*-splicing, overlapping or “hybrid” techniques) have been used to express the *MYO7A* and the *ABCA4* genes (both with CDS over 6 kb in length and expressed in the retina) and showed effective *in vitro* protein expression (Dyka et al., 2014). Different studies have also shown that these dual AAV approaches are efficient at transducing mouse and pig retinas, with the *trans*-splicing and hybrid systems being the most efficient *in vivo* (Colella et al., 2014; Dyka et al., 2014; Trapani et al., 2014, 2015).

Even though the dual AAV method feasibility has been demonstrated by rescue of disease phenotype in different models, a more fundamental understanding of vector reconstitution efficiencies has been lacking. Furthermore, despite promising results, the low efficiency of AAV dual strategies remains a hurdle for further ocular translational applications, even when using vectors with extremely high retinal transduction capacity like AAV8. For this purpose, we generated a dual vector reporter construct approach based on quantification of β-galactosidase enzymatic activity, which offers better precision than quantification based on western blot band intensity comparisons. Further, an enzymatic assay identifies reconstitution based on protein function, not just size, thus excluding potential incorrect reconstitution events. Our aim in this study was to offer a more systematic approach to dual strategy comparisons *in vitro* and *in vivo* using a unique oversized reporter construct and to test different variables that could be behind the low efficiency reported when using AAV dual approaches.

## Materials and methods

### Constructs, vectors, and AAV production

The oversized Rainbow transgene construct was synthesized by DNA2.0 (Newark, CA) and cloned into an ITR containing plasmid. All dual left and right and control CMV.*lacZ* constructs were then derived from the original oversized Rainbow with extra features (sD and sA sites, *AP* sequence) added via PCR and standard restriction enzyme cloning. AAV2/2, AAV2/8, and AAV2/Anc80L65 for dual and control constructs were prepared at Gene Transfer Vector Core (vector.meei.harvard.edu/) at Massachusetts Eye and Ear Infirmary as previously described (Zinn et al., 2015).

### *In vitro* experiments

All *in vitro* experiments were performed on HEK293 cells on poly-D-lysine-coated 6-well (Sigma, Natick, MA) plates for plasmid transfections and 96-well plates (Sigma, Natick, MA) for AAV transduction experiments. Cells were passaged as standard and plasmid transfected with equal molarities or AAV transduced at around 70% confluency. Transfection experiments were done in triplicates with identical conditions to ensure equal transfection efficiencies. AAV particles were added directly to the cell media at the desired MOI while plasmid transfection was performed using polyethylenimine (Polysciences Inc., Warminster, PA). Cells were then incubated for 48 h at 37°C with 5% CO_2_ after which media was aspirated and proceeded to β-galactosidase detection method as described below in Section Protein Assay.

### *In vivo* experiments

Wild-type C57BL/6J-Tyrc-2J/J male mice (6–8 weeks old) were purchased from the Jackson Laboratory (Bar Harbor, ME) and kept at the Schepens Eye Research Institute (SERI) Animal Facility. All animal procedures were performed in accordance with protocols approved by the institutional animal care and use committees at SERI and conformed to the guidelines on the care and use of animals adopted by the Association for Research in Vision and Ophthalmology (Rockville, MD).

#### Subretinal injections

Subretinal injections were performed by injecting 2 μl of vector between the retinal pigment epithelium (RPE) and photoreceptor layer by scleral tunnel approach, using the Micro4 injector system and 10 μl Nanofil syringe (World Precision Instruments, LLC., Sarasota, FL). For the combination of left (L) and right (R) constructs (low dose group), viral genomes (vg) were diluted together to generate a final titer of 1.5E12 vg/ml (total 3E9 vg/eye). For the high dose group, equal volumes of R and L constructs were mixed together at the concentration of 1.5E13 vg/ml, which generated a final titer of 7.5E12 vg/ml (1.5E10 vg/eye) of each construct. As a result, the “high dose” group received 5 times more vg than the “low dose” group. Monocistronic CMV.*lacZ* vectors were injected with half of total L + R dose so that a theoretical 100% reconstitution would result in identical full genome amounts as compared to control. Finally, the left construct alone was injected at a concentration of 1.5E12 vg/ml (3E9 vg/eye). Each separate experiment used 4–6 eyes per experimental group. Eyes were collected 4 weeks post-injection for RNA or protein isolation by use of AllPrep DNA/RNA/Protein kit (Qiagen, Hilden, Germany) or Galacto-Light Plus™ System (Applied Biosystems, Foster City, CA), respectively.

#### Real-time PCR

Real-time PCR was performed in duplicate to quadruplicate using the TaqMan® gene expression master mix (Thermo Fisher Scientific, Waltham, MA) on an ABI 7500 Real Time PCR System (Applied Biosystems). RNA was isolated as described above and reverse-transcribed using the QuantiTect Reverse Transcription Kit (Qiagen). Relative transcript levels were assessed using the ΔCT method with GAPDH as the reference gene (Taqman Assay Mm99999915_g1; Cat. # 4331182; Life Technologies, Woburn, MA). Primers used for *lacZ* detection were the following (5′–3′): GATCAAATCTGTCGATCCTTCC (forward) and GCGTACATCGGGCAAATAAT (reverse) along with Universal Library probe #70 from Roche (Basel, Switzerland). The *lacZ* primer/probe assay was specifically designed to span the exact location where the *lacZ* gene is split into left and right constructs in the dual hybrid strategy. Real Time PCR figure presented represents the average of two independent plates, each containing four eyes per group.

### Protein assay

**Figure 1:**
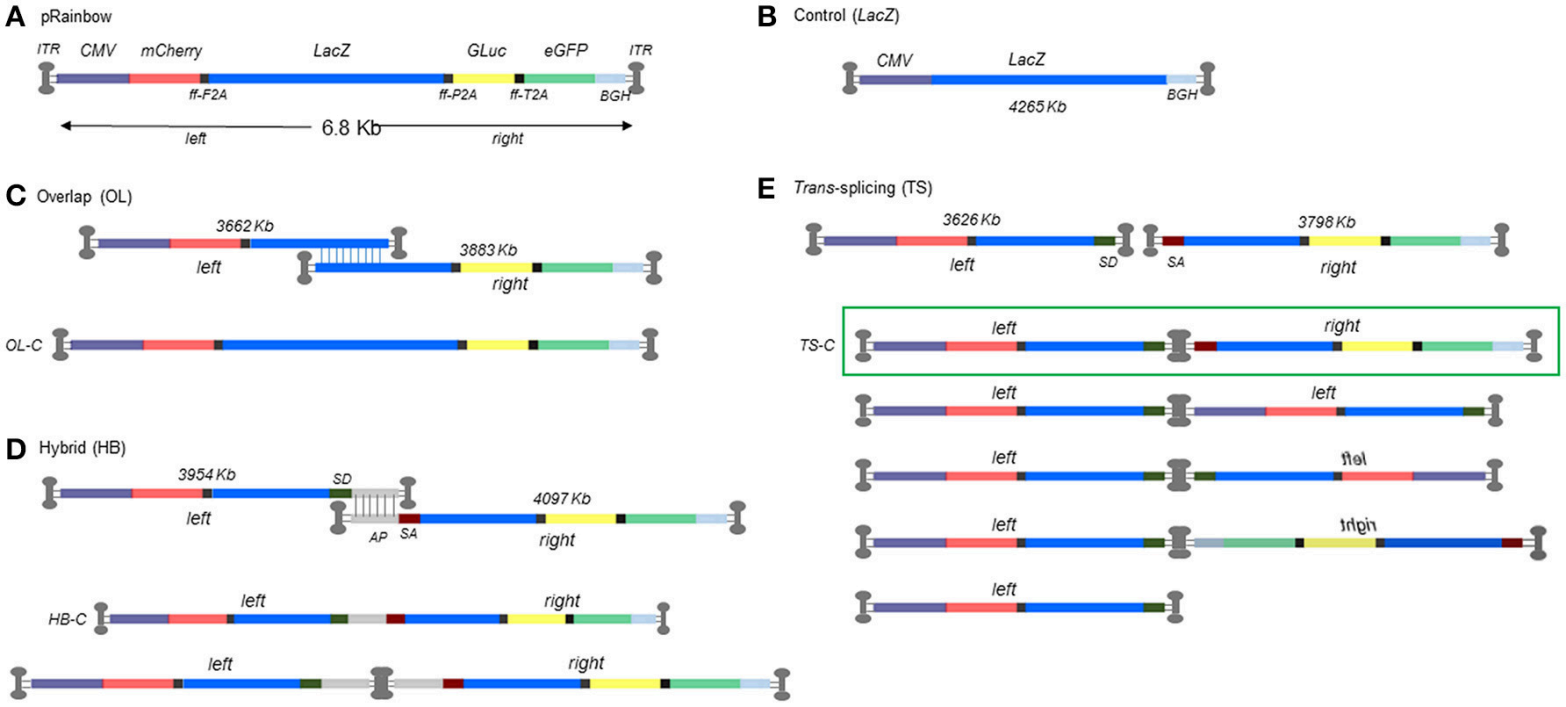
Schematic representation of dual AAV strategies with size of each left and right construct. **(A)** Oversized monocistronic Rainbow construct showing order of reporter genes separated by furin-2A peptide cleavage sites. **(B)** Normal sized control construct (CMV.*lac*Z). **(C)** Overlap (OL) dual AAV strategy showing split left and right constructs and reconstituted genome structure. **(D)** Hybrid (HB) dual AAV strategy (top) and the equivalent reconstituted genome structures underneath. **(E)** *Trans*-splicing (TS) dual AAV strategy (top) and overview of the possible concatameric permutations for TS vectors with the highlighted combination (green box) that would lead to a correctly reconstituted genome. OL-C, HB-C, and TS-C denominate the structure of the control plasmid used in Figure 2A.

**Figure 2:**
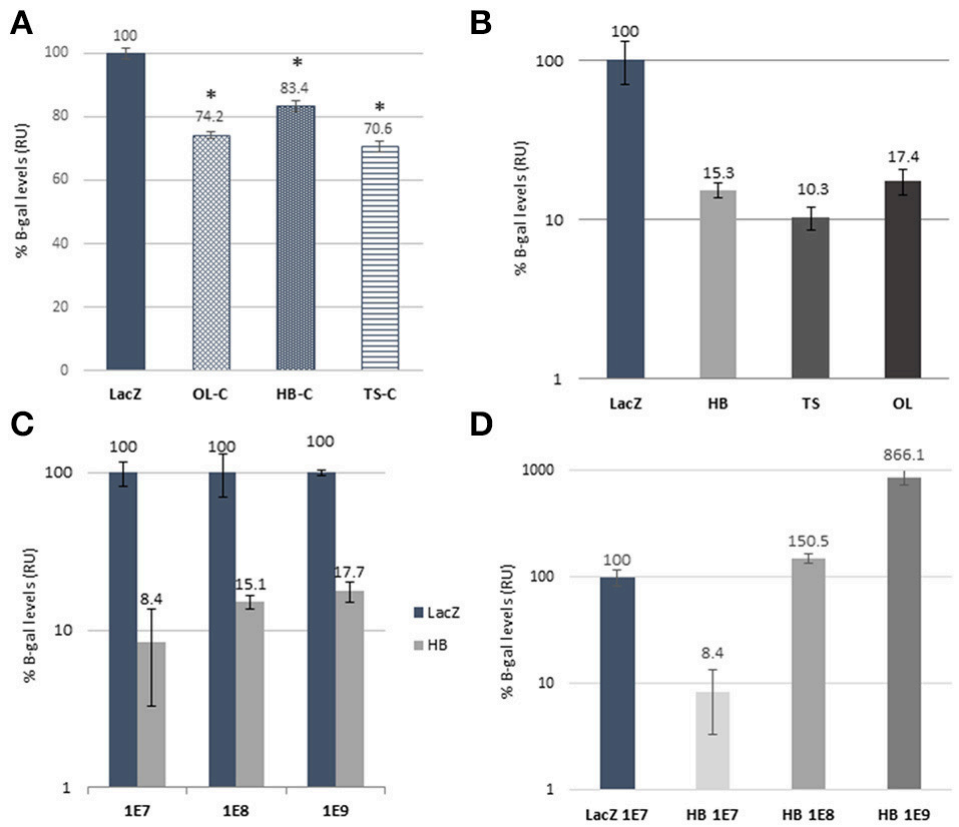
*In vitro* validation of dual AAV strategies. **(A)** Comparison of expression levels of β-galactosidade from plasmid transfection of different iterations of reconstituted Rainbow constructs (OL-C, HB-C, and TS-C) and CMV.*lacZ* coding control plasmid (LacZ). Two tailed Student's *t*-test was used to determine significance compared to CMV.*lacZ* (^*^p < 0.01). Error bars represent S.E.M. **(B)** *In vitro* efficiency comparison of AAV2 dual hybrid (HB), *trans*-splice (TS) and overlap (OL) to monocistronic CMV.*lacZ* (LacZ) construct of β-galactosidase protein expression. **(C)** Dose experiment comparison of AAV2 HB dual constructs at three different doses (MOI of 1E7, 1E8, and 1E9) with each dose relative to CMV.*lacZ* at the equivalent dose while **(D)** shows expression levels of the three doses of HB relative to CMV.lacZ control at the 1E7 dose. *In vitro* data are represented as mean ± S.D. from 1 to 2 assays done in triplicates and values are shown as percentage of control (CMV.*lacZ*) after background subtraction (L construct only) and corrected for plasmid reconstitution rates (using data from graph A).

Both *in vivo* and *in vitro* β-galactosidase quantification were performed using the Galacto-Light Plus™ System (Applied Biosystems) and a Synergy H1 Hybrid l Reader luminometer plate reader (BioTek, Winooski, VT). *In vitro* experiments followed the Direct Lysis Protocol for Microplate Cultures as described by the manufacturer. *In vivo* experiments followed a similar protocol but with the following modifications. Whole retinas (minus RPE) were dissected from injected eyes at 4 weeks post-injections and added to 100 μl of T-PER Tissue Protein Extraction (Thermo Fisher Scientific) and followed manufacturer's protocol for protein extraction. Protein extracts were then quantified using the standard protocol from the Coomassie Plus™ (Bradford) Assay Kit (Thermo Fisher Scientific) and 10 μg of total protein lysate was then used for β-galactosidase quantification with the Galacto-Light Plus™ System. Quantification of relative β-galactosidase levels in each experiment was done by first subtracting from the raw values a baseline measurement (from the same plate) of an R- or L-only construct well/eye. These values were then normalized to a CMV.*lacZ* construct of the same MOI/titer. Based on our plasmid transfection experiments β-galactosidase expression from Rainbow plasmids (original intact and hypothetical reconstituted plasmids containing introns, as indicated in Figure 1) was lower than from CMV-*lacZ* plasmid (Figure 2A). Therefore, even 100% reconstitution efficiency of e.g., hybrid DUO vectors could only result in 83.4% protein levels of CMV-*lacZ* control. To account for this discrepancy all normalized values were further divided by fold reduction in expression of each respective control plasmid, as indicated by values in Figure 2A.

### Statistical analysis

A two-tail student's *t*-test was performed to analyze all data sets. The data was considered significantly different when *p* < 0.01. Error bars represent standard error mean (S.E.M.) or standard deviation (S.D.) as indicated.

## Results

### Generation of dual vector reporter constructs

In order to evaluate the efficiency of dual AAV vector strategies, we designed an oversized monocistronic multiple reporter construct that would only allow expression of each and all of the components following expected reconstitution of the oversized cistron. The construct illustrated in Figure 1A comprised of *mCherry, lacZ, Gluc*, and *eGFP* coding sequences separated by furin-2A peptide cleavage sites. The full-length construct, named pRainbow, is expressed from a CMV promoter and translated into a single polypeptide which is subsequently cleaved to separate the four reporter proteins. Dual vectors were constructed by splitting pRainbow into two parts (*left* i.e., 5′ and *right* i.e., 3′) within the *lacZ* sequence. Reconstitution strategies were designed to rely on homologous recombination based on sequence homology (overlap; OL), AAV splicing across ITRs of a concatamerized dimeric genome (*trans*-splicing; TS), and combination of the two (hybrid; HB) (Figure 1). Each approach will result in reconstitution of transcriptionally active Rainbow construct. OL constructs of three different lengths of sequence homology within the *lacZ* gene were generated and tested (1,100, 600, and 300 bp, data not shown). The construct containing the largest homology sequence generated the best reconstitution efficiency therefore was used hereafter (data not shown). Figure 1E shows the different possible permutations for TS constructs, highlighting the only combination that would lead to correct genome reconstitution.

As neither left nor right constructs contains a full *lacZ* coding sequence, correct reconstitution can be accurately quantified by measuring the amount of its protein product, β-galactosidase, with standard enzymatic assays. Since our interest was to assess the relative efficiency of a dual AAV (OL, TS, or HB) vs. the traditional single AAV approach, we first wanted to account for the inherent difference in expression level due to the distinct expression contexts for *lacZ* of the single vs. each of the dual AAV *lacZ* expression cassettes. Original Rainbow sequence is anticipated to be restored only after OL reconstitution, whereas TS will have an intron containing an AAV ITR sequence, and HB will have an intron with AP or AP-ITR-AP sequence (Figures 1D,E). Therefore, we measured β-galactosidase expression from cells transfected with equimolar amounts of plasmids coding for the different iterations of reconstituted Rainbow plasmid constructs from OL, TS and HB or CMV.*lacZ* coding control plasmid. The results show that all possible Rainbow construct combinations are capable of reconstituting with β-galactosidase levels between 70.6 and 83.4% of the monocistronic CMV.*lacZ* control (Figure 2A), illustrating that the *lacZ* expression from a Rainbow-type cassette was high and only moderately decreased compared to an expression cassette that expresses only *lacZ*, outside of the other reporter genes. All protein expression data from dual vector reconstitution efficiencies were normalized against CMV.*lacZ* controls at the same MOI unless otherwise stated, and then corrected for β-galactosidase levels using the respective plasmid control data shown in Figure 2A as follows: OL transductions were divided by 0.74 (OL control, OL-C), HB were divided by 0.83 (HB control, HB-C) and TS were divided by 0.71 (TS control, TS-C).

### Reconstitution efficiencies of dual vectors *in vitro*

**Figure 3:**
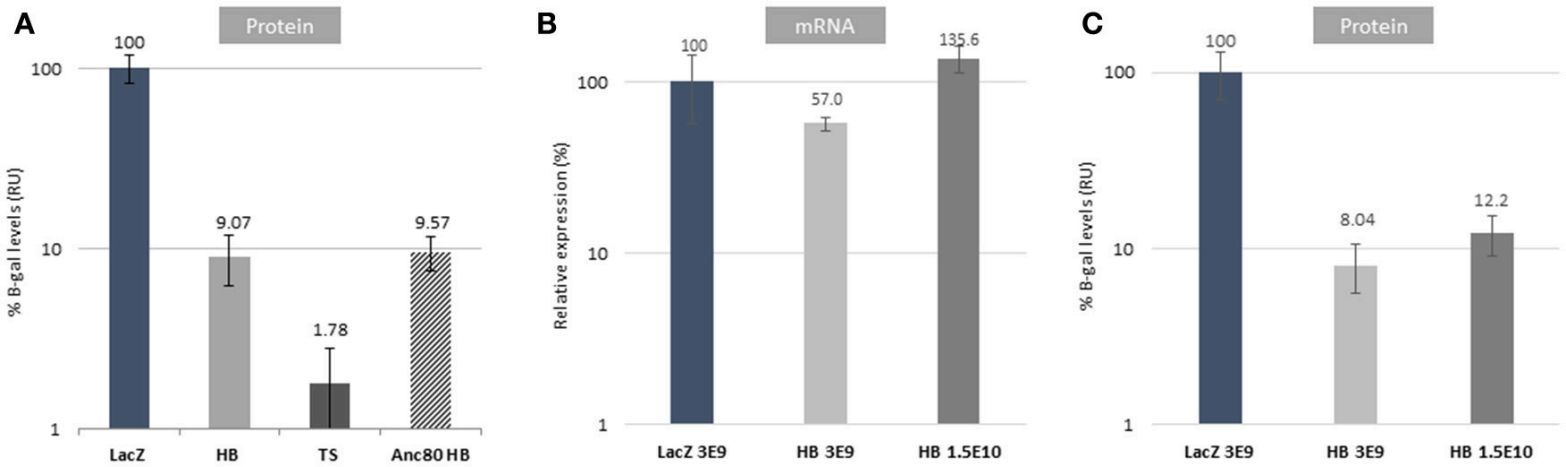
*In vivo* validation of dual AAV strategies. **(A)** *In vivo* comparison of dual strategies after subretinal injections using AAV8 or Anc80_L65 relative to AAV8 *CMV.lacZ* (LacZ) control at a dose of 3E9 vg/eye. **(B)** mRNA expression data comparing reconstituted genomes from HB at different doses (low: 3E9 vg/eye; high: 1.5E10 vg/eye). The delta-CT presented is normalized to GAPDH, relative to *CMV.lacZ* (LacZ). **(C)** Protein expression levels of HB *in vivo* at different doses (low: 3E9 vg/eye; high: 1.5E10 vg/eye). *In vivo* data are represented as mean ± S.E.M. from 2 to 3 assays done in duplicate to quadruplicate from 4 to 6 eyes per group.

Next, we compared efficiencies of different dual vector strategies *in vitro*. HEK293 cells were transduced with *left* and *right* AAV2/2 dual vectors and CMV.*lacZ* control vector. No β-galactosidase was detected in cells transduced with left or right vectors by themselves (data not shown). Total doses for dual vector combinations were twice to control so that theoretical maximum copy number of reconstituted Rainbow would be equal to control. Based on β-galactosidase levels, reconstitution efficiencies were 10.3, 15.3, and 17.4% of control for TS, HB, and OL, respectively (Figure 2B). OL reconstitution is dependent on overlapping sequence length, with shorter overlap showing lower efficiencies (data no shown), and on the recombinogenic capacity of this sequence, which is expected to be gene-dependent. As such, the OL strategy would result in different reconstitution efficiencies for every transgene. In contrast, reconstitution based on uniform homologous sequences, such as in the hybrid approach, should result in equal efficiencies regardless of the transgene. Therefore, due to homology sequence-specificity and undetectable *in vivo* reconstitution of OL vectors (Figure 3A), further *in vitro* characterization was performed with HB constructs only.

Effect of dose on reconstitution was studied by transducing HEK293 cells with increasing amounts of HB dual vectors. The data show moderately increasing levels of β-galactosidase (8.4%, 15.1% and 17.7% for 1E+7, 1E+8 and 1E+9 vg, respectively) when compared to matching control doses (Figure 2C) indicating increased co-transfection due to higher MOIs. Further, increasing dual vector doses 10- and 100-fold (from 1E7 to 1E8 and 1E9) we see corresponding 18- and 103-fold increases in β-galactosidase levels, respectively when compared to 1E7 control (Figure 2D).

### Reconstitution efficiency of dual vectors *in vivo*

Dual vector reconstitution efficiencies were evaluated *in vivo* in the mouse retina. C57BL/6J-Tyrc-2J/J mice were injected subretinally with AAV2/8 left and right dual vectors combinations and control CMV.*lacZ* vectors. Total protein was extracted from dissected retinas and β-galactosidase levels were measured. Reconstitution efficiencies were 9.07 and 1.78% of control for HB and TS, respectively (Figure 3A). OL reconstitution efficiency (1,100 bp homology construct) was below assay background level and is therefore not shown (−0.67% ± SEM 0.54). Further *in vivo* experiments were only performed with HB vectors, as it proved to be distinctly the most efficient strategy.

Next, we studied the effects of capsid serotype on protein reconstitution efficiency. We chose to compare AAV2/8 and Anc80L65 vectors, as Anc80L65 has been shown to transduce retinal cells with a higher efficiency than AAV8 (Zinn et al., 2015). As shown in Figure 3A, retinal cell transduced with Anc80L65 had an almost identical level of HB reconstitution compared to AAV8 (9.57%) even though a comparison of the absolute levels of β-galactosidase from the control CMV.*lacZ* vector for each serotype shows that Anc80L65 is 14-fold higher than AAV8 (data not shown).

Our final experiment investigated reconstitution efficiency and effect of vector dose on protein and mRNA levels (Figures 3B,C). Interestingly mRNA levels (Figure 3B) showed significantly higher rates of reconstitution than protein levels (Figure 3C), however a 5-fold increase in vector dose only resulted in 2.2- and 1.5-fold increases in mRNA and protein levels, respectively. All mice injected with left construct alone did not have LacZ expression as detected by the qPCR assay: CT values “not determined” or 36.2+ (data not shown).

## Discussion

Gene replacement therapies utilizing AAV vectors have proven their potential in treating several diseases with limited options for alternate treatments. The first commercial AAV gene therapy product has already seen the light of day (Gaudet et al., 2016), and promising results from numerous ongoing clinical trials may herald the emergence of other products in the near future (Naldini, 2015; Pierce and Bennett, 2015). However, despite its safety and efficacy as a gene therapy vector, AAV has its shortcomings, one being the limited capacity of its genetic payload (Kotterman and Schaffer, 2014). Even though, in humans, the median protein size is only 375 amino acids long (Grieger and Samulski, 2005), easily fitting into an AAV vector with suitable elements for regulation of expression, several important candidate genes for gene replacement therapies, such as *USH2A* (Usher syndrome type 2A), *F8* (hemophilia A) or *DMD* (Duchenne muscular dystrophy), are too large. Different strategies to enable AAV gene therapies for larger genes have been studied, including gene truncation, *in vivo* genome editing, and use of dual AAV vectors. Each strategy comes with their own potentials and problems.

Dual AAV vector strategies rely on rAAV genome concatamerization utilizing ITRs and/or transgene cassette sequence homologies. Vector reconstitution has been reported *in vitro* in several cell lines, and *in vivo* in mouse liver, skeletal muscle, and retina (Grieger and Samulski, 2005). Depending on experimental details, *in vivo* efficiencies of ~5–10% as compared to transgene expression from single AAV approaches have been reported, and in some model systems this has resulted in rescue of a disease phenotype, indicating that efficiencies with clinical relevance can be achieved. However, even though dual AAV vector strategies have been shown to work, details behind cellular mechanisms underlying vector reconstitution are still poorly understood. Despite the seemingly simple premise of dual AAV strategies, there are several conceptual inefficiencies that could affect reconstitution efficiency and would need to be overcome when considering clinical applicability and broader applications. These include not only general parameters that affect all dual strategies, but also ones specific to each strategy. Amongst the general issues, it is thought that (i) co-transduction efficiency and (ii) efficiency of expressing a large transgene cassette, play a major role in determining overall reconstitution levels and need to be taken into consideration in the experimental design (e.g., choice of serotype, expression system etc.). However, when analyzing the different dual strategies, the parameters to consider become substantially more complex. As we show in Figure 1E, there are several different ways that the *left* and *right* vectors from the TS approach could come together, which overall lowers the probability of correct concatamerization and consequently reconstitution efficiency. For both the OL and HB approaches, the homology sequence will be critical in determining the levels of reconstitution. In the OL strategies, sequences will be gene-specific dependent with only a few variables like overlap size and sequence optimization amenable to modifications. For HB, however, the discovery of better homology sequences could help improve reconstitution levels and efficiency.

Studies comprehensively comparing efficiencies of different dual AAV vector strategies in the retina have been limited. Even though vector reconstitution in the retina has been detected for clinically relevant large transgenes, such as *ABCA4* or *MYO7A*, due to lack of proper controls (e.g., using oversized monocistronic vectors) and accurate protein quantification methods, determining parameters for clinical approaches has been lacking (Dyka et al., 2014; Trapani et al., 2014, 2015). Here, we compared efficiencies of commonly used dual vector strategies (TS, OL and HB) *in vivo* in mouse retina using an oversized ubiquitously expressed *lacZ* reporter construct. Our reporter construct was designed with two main purposes; (i) to provide an accurately quantifiable protein product and (ii) to allow generation of normal sized monocistronic control vectors. Functionality of our dual vectors and controls was assessed *in vitro*, and showed that protein expression from oversized reporter was similar to normal sized controls. When reconstitution efficiencies based on protein expression were quantified by normalizing *lacZ* expression levels to their respective controls, levels of 15.3, 17.4, and 10.3% were detected for HB, OL and TS, respectively. Interestingly, different relative efficiencies have been reported by others, especially regarding HB and TS (Dyka et al., 2014; Trapani et al., 2014). While all designs seemingly use sequences originally reported by Ghosh et al. (2008), the drastic differences in HB reconstitution within *AP* intron sequence seen between the different studies, may reflect undetected or unreported mutations between sequences, especially in the ITRs, indicating sensitivity of reconstitution to minor variations (Ghosh et al., 2008). We also studied whether the effect of dose of dual vector transduction correlates linearly with reporter transgene expression *in vitro*, and detected 17 and 102-fold increases in expression when dual vector dose was increased 10 and 100-fold to controls, respectively. When compared to controls of equal dose, reconstitution efficiencies increased from 8.4 to 15.1 to 17.7% for doses of 1E7, 1E8, and 1E9, respectively. The increase in efficiency likely reflects increased vector co-transduction efficiency. However, at doses of 1E8 and 1E9 co-transduction efficiency approaches 100%, and the minor increase in reconstitution efficiency suggests that reconstitution is not significantly dependent on intracellular rAAV vector genome amounts.

Interestingly, our data indicates different relative efficiencies of reconstitution *in vivo* in mouse retina with levels compared to control, of 9.07% for HB, 1.78% for TS, and below detection for OL. The drastic decrease in OL efficiency may reflect differences in activities of pathways responsible of homologous recombination *in vivo* as compared to *in vitro*. Even though others have detected OL reconstitution in RPE cells, but not photoreceptors, this may be due to differences in overlap lengths and sequences (Trapani et al., 2015). Effect of serotype on vector reconstitution was studied by comparing AAV8 and Anc80L65, both of which transduce retinal cells with high efficiencies (Zinn et al., 2015). Detected reconstitution efficiencies were similar. Further, our *in vivo* dose escalation study only resulted in moderate increase in protein levels. With similar observations made from our *in vitro* dose escalation study, taken together these data support our conclusion that once efficient co-transduction is achieved, further increases in reconstitution efficiencies are not strongly dependent of vector genome amounts. Therefore, these studies may suggest an inherent limit for vector reconstitution after dual AAV delivery. However, data from us and others also indicates that these efficiencies depend on transgene sequences, and may possibly be increased by using more recombinogenic overlap and/or ITR sequences (Yan et al., 2005).

Surprisingly, our data revealed a major discrepancy between levels of reconstituted protein and mRNA *in vivo*. While reconstitution efficiency was assessed to be ~10% based on protein activity, mRNA amounts suggested ~60% efficiency. While our *in vitro* data supports ~10% reconstitution efficiency, as do earlier studies by others, the full story may be more complicated. Both transcription and translation are strongly affected by exact DNA and mRNA sequences, respectively, as well as their cellular environments (Sonenberg and Hinnebusch, 2009; Alpert et al., 2017). In particular, mRNA length has been shown to negatively correlate with stability, which would explain our observations (Feng and Niu, 2007). Assessing dual AAV vector reconstitution quantitatively based on mRNA or protein levels may thus be an inherently flawed approach, as proper controls for these experiments are not feasible. Rate of rAAV genome reconstitution may be quantifiable, but resulting mRNA or protein levels are not, unless controls of identical sequences are used. As dual AAVs are studied in the context of oversized transgenes, oversized monocistronic controls cannot be used. However, as being oversized is not a prerequisite for use of dual vector strategies, mechanics and efficiencies of vector reconstitution could also be studied with transgenes that fit the AAV. Studies of this nature could be informative in learning more about basic AAV biology, and help design better dual vector strategies. For clinical purposes, dual AAV approaches may be feasible if sufficient protein expression levels to affect the disease phenotype can be achieved, regardless of the *de facto* efficiency of the reconstitution process.

An alternate method for expressing large proteins relying on intein protein sequences has also been reported (Li et al., 2008; Truong et al., 2015; Chew et al., 2016). In this approach, the transgene is divided into two parts in which 5′ half of the gene will express a C-terminal intein sequence, and 3′ half an N-terminal intein sequence. Reconstitution will therefore take place on peptide level in a process termed protein splicing, where the intein sequences excise themselves and join the remaining peptides together. Even though proof of concept studies have shown feasibility of this approach, its efficiency has never been thoroughly characterized. Further, proteins can be split by inteins only at certain sites, and the reconstituted protein may not reach proper conformation due to the two peptides folding separately. Also, as the two peptides need to be expressed, both vector halves will need their separate transcriptional regulators, and with the added length of the intein sequences, gain of payload carrying capacity is diminished as compared to genome reconstitution approaches. Finally, inteins are of bacterial origin, and as such may be immunogenic. However, despite these drawbacks, intein mediated dual vector systems may warrant further studies.

The *in vivo* aspect of this project focused on targeting the retinal pigmented epithelium and the photoreceptor cells by subretinal injection to maximize co-transduction efficiencies and thus vector reconstitution. However, co-transduction can also be achieved by intravitreal injection, which targets the retinal ganglion cells and inner retina (Hung et al., 2016), albeit at much lower efficiencies than by subretinal injections. With the increasing focus on modified capsids to augment retinal transduction, future experiments may employ an intravitreal injection that can reach the photoreceptor cells, thereby reducing the need for an invasive subretinal injection (Boye et al., 2016).

In conclusion, our work provides quantitative data on vector reconstitution efficiencies *in vivo* in the retina. When targeting retinal cells that are efficiently transduced by AAV, protein expression levels of ~10% of monocistronic vector levels may be achieved. However, this may not be a reliable indicator of actual reconstitution efficiency. Our work may offer insight on developing improved dual vector approaches. As of now, dual AAV vector strategies may be of use in gene therapy modalities where even limited transgene expression has positive effects on disease phenotype.

## Author contributions

LC, HT, and SW contributed to the experimental design, performed experiments, data analysis and manuscript writing. LV led the experimental design and final drafting of the manuscript. RX and ML performed experiments and JB contributed to experimental design.

### Conflict of interest statement

LV holds founder equity in GenSight Biologics, is a consultant to a number of biotech and pharmaceutical companies, and is an inventor on gene therapy patents, including Anc80L65. The other authors declare that the research was conducted in the absence of any commercial or financial relationships that could be construed as a potential conflict of interest.
